# Are students less likely to respond to routinely delivered psychological treatment? A retrospective cohort analysis

**DOI:** 10.1016/j.comppsych.2022.152348

**Published:** 2022-11

**Authors:** Phoebe Barnett, Rob Saunders, Joshua E.J. Buckman, Ana Cardoso, Mirko Cirkovic, Judy Leibowitz, Nicole Main, Syed A. Naqvi, Satwant Singh, Joshua Stott, Lila Varsani, Jon Wheatly, Stephen Pilling

**Affiliations:** a Centre for Outcomes Research and Effectiveness, Research Department of Clinical, Educational, & Health Psychology, University College London, London, UK; b National Collaborating Centre for Mental Health, Royal College of Psychiatrists, London, UK; c iCope - Camden & Islington Psychological Therapies Services, Camden and Islington NHS Foundation Trust, London, UK; d North East London NHS Foundation Trust (NELFT), London, UK; e Talk Changes: City & Hackney IAPT Service, Homerton University Hospital NHS Foundation Trust, London, UK; f Let's Talk IAPT–Barnet, Enfield & Haringey Psychological Therapies Service, Barnet, Enfield & Haringey Mental Health Trust, London, UK; g Waltham Forest Talking Therapies–North East London Foundation Trust, Thorne House, London E11 4HU, UK; h Ageing, Dementia And Psychological Therapies (ADAPT) Lab, Research department of Clinical, Educational, & Health Psychology, University College London, UK; i Camden and Islington NHS Foundation Trust, London, UK

**Keywords:** Student, Cohort, Depression, Anxiety, Mental health, Psychological treatment

## Abstract

**Background:**

Depression and anxiety disorders are increasingly prevalent among university students, making the provision of effective treatment in this population a priority. Whilst campus-based services provide some psychological treatments, many students are treated by routine adult psychological treatment services which have no focus or adaptations to treatment for student populations. We aimed to compare psychological treatment outcomes between university students and young adults (aged 18–25) in employment to explore whether routinely delivered psychological interventions are equally effective for these groups, or whether students report poorer outcomes.

**Methods:**

A retrospective cohort was formed of 19,707 patients treated by eight National Health Service (NHS) Improving Access to Psychological Therapies (IAPT) services in England. Associations between student status (compared to same-age employed adults) and psychological treatment outcomes were explored using logistic regression models. Models were adjusted for important treatment, clinical and demographic characteristics, and propensity score matching was used to explore the robustness of effects.

**Results:**

Students and the employed comparison group were similar on baseline characteristics at assessment, but students were less likely to reliably recover (OR = 0.90 [95% CI = 0.83;0.96]) and reliably improve (OR = 0.91 [95% CI = 0.84;0.98]) by the end of treatment in fully adjusted models. Students and the employed group did not differ regarding the likelihood of deterioration (OR = 0.89 [95% CI = 0.78;1.02]) or treatment dropout (OR = 1.01 [95% CI = 0.93;1.11]).

**Conclusions:**

Students appear at risk of poorer outcomes compared to employed younger adults when treated in routine psychological treatment services. Students may require additional support and treatment adaptations that account for student-specific stressors as this might improve psychological treatment outcomes.

## Highlights

• Students in this analysis were less likely to reliably recover or improve following routine psychological service treatment
• The students were also no more likely to reliably deteriorate or drop out of treatment compared to employed controls
• Treatment intensity, frequency, or medium did not moderate findings
• Adaptation of care pathways and content to meet student specific needs and may benefit students.

## 1 Introduction

Mental health conditions are prevalent among students in higher education, with as many as one third of all first-year students reporting an anxiety, mood or substance use disorder [1,2]. There are mixed findings around whether students have more mental health problems than their age matched peers [3], and many of the demands of young adulthood, regardless of student status could lead to increased risk of mental health problems [4,5]. However, many of the other demands typically placed on students, for example separation from support networks as a result of moving away from home, additional financial burden, increased alcohol and drug use and academic stress may contribute to heightened risk of these conditions compared to non-students [[6], [7], [8], [9], [10], [11]]. Given that these stressors are associated with poorer mental health treatment engagement and outcomes [[12], [13], [14]], it is unsurprising that psychological distress remains high throughout time in higher education [15].

There have been efforts to improve university campus-based mental health services in recent years [16,17], however a large number of students are seen in routine healthcare services external to student campuses [18,19]. This is likely to continue to be the case given long waiting times and limited choice of treatment in some campus-based services [16,18]. Further, universities may consider general student wellbeing as their responsibility and as such tailor campus based services to meet this need [20,21], but consider healthcare and illness (including the experience of mental health conditions) as the responsibility of external medical services. Consequently, those students experiencing the most severe symptoms may be seen outside of campus-based support. However, it is not clear how well such services meet the needs of students in particular and there has been limited explicit comparison of treatment outcomes in external metal health services between students and non-students. This contrasts with research with explicit focus on the services provided within universities, which have received significantly more scrutiny in recent years [18,22]. Comparisons within external services may help to inform service design and provision to make it better tailored to student needs, particularly given calls for improved links between university based and national health service mental health services as part of a whole university approach [23,24]. This is important as students may be more likely to disengage from treatment [25], and there are a number of contextual issues specific to the student population such as changes to residence and term-time only availability which may result in poorer outcomes [26].

Similarly aged adults who are in employment constitute a suitable group to compare to students, as people who are neither in education or employment (those who are NEET) have particularly poor outcomes [27,28]. For example, findings from previous research which suggest that students may fair better than age-matched peers [3] have not differentiated between employed and unemployed controls, making it hard to establish where on the spectrum of vulnerability university students may sit. This study therefore explores the differences in characteristics and outcomes for students and same-age employed adults who were treated in psychological treatment services as part of routine healthcare provision (i.e. not campus-based care), and examines potential treatment moderators of outcome.

## 2 Materials and methods

### 2.1 Services

Patients who attended the Improving Access to Psychological Therapies (IAPT) services that are part of the “North Central and East London IAPT Service Improvement and Research Network” (NCEL IAPT SIRN) [29] formed the dataset for this analysis. IAPT services are part of the English National Health Service (NHS) and include both primary care and community-based mental health services operating across England with over 1.5million referrals annually [30]. They deliver evidence-based psychological therapies primarily for depression and anxiety disorders within a stepped-care model, in line with UK national guidelines [31]. Within IAPT services, sessional outcome measurement is mandated, which means that pre- and post- intervention data are available for more than 98% of episodes [32].

### 2.2 Participants

The initial sample consisted of 483,683 participants who were referred to NCEL services from August 2008 to August 2020. Participants meeting the following criteria were included in the analysis: aged between 17 and 25 at time of referral, as 70% of students who enrol in higher education are within this age bracket [33], better allowing for age-matched pairing; had completed treatment, at assessment met the clinical criteria for “caseness” on any depression or anxiety symptom measures used by the services, and reported being either a) a student undertaking full or part-time study who are not working or currently seeking work or b) employed at their initial assessment. Participants were also required to have attended at least two treatment sessions and completed outcome measures at those sessions in order to calculate study outcomes. Two sessions of treatment are considered to be the minimum number for an episode of care in these routine healthcare settings [34].

### 2.3 Measures

The measures collected within IAPT services relevant to this study along with relevant thresholds are reported in Table 1.

**Table 1:** Measures.

Item	Questionnaire	Additional Information/thresholds
*Baseline mental health symptoms*		
Depressive symptoms	Patient Health Questionnaire 9-item version (PHQ-9; [[55], [56]])	Scores of 10 or above on the PhQ-9 indicate cases of depression, while a change of 6 or more indicates reliable change.
Anxiety symptoms	The Generalized Anxiety Disorder Scale 7-item version (GAD-7; [60])	Scores of 8 or above on the GAD-7 indicate cases of generalized anxiety while a change of 4 or more indicates reliable change.
	“Anxiety disorder specific measures” (ADSMs)	ADSMs are used in place of the GAD-7 if a specific anxiety disorder is identified as the main problem.
	1. *Agoraphobia: Mobility inventory (Chambless, Caputo, Jasin, Gracely, & Williams, 1985)*	Scores of 2.3 and above indicate cases for agoraphobia, while a change of 0.73 indicates reliable change
	2. *Health Anxiety: Health Anxiety inventory* [58]	Scores of 18 and above indicate cases of health anxiety, while a change of 4 indicates reliable change
	3. *Obsessive Compulsive Disorder (OCD): Obsessive Compulsive inventory* [54]	Scores of 40 and above indicate cases of OCD, while a change of 32 indicates reliable change
	4. *Panic Disorder: Panic Disorder Severity Scale (PDSS;* [59]*)*	There is no threshold for indicating cases or reliable change for the PDSS. Therefore IAPT outcomes for individuals with Panic Disorder are calculated using the GAD-7.
	5. *Post-Traumatic Stress Disorder (PTSD): Impact of events Scale (IES-R;* [53]*)*	Scores of 33 and above indicate caseness for PTSD, while a change of 9 indicates reliable change
	6. *Social Anxiety Disorder: Social Phobia Inventory* [61]	Scores of 19 and above indicate caseness for social anxiety disorder, while a change of 10 indicates reliable change
Phobic anxiety	IAPT Phobia scales (IAPT, 2011; [30])	The phobia scales are three questions which assess the extent that a person avoids situations related to agoraphobia, social phobia and specific phobia.
“Problem descriptor”	Probable or confirmed diagnosis using ICD-10 codes	Used to match participants based on presentation symptoms to evidence-based treatment protocols. Categorised following previous studies (Buckman et al., 2018; Saunders et al., 2021) as depression; mixed anxiety and depression; generalized anxiety disorder; OCD; PTSD; and phobic anxiety or panic.

*Functional and Social impairment*		
Personal functioning	The Work and Social Adjustment Scale (WSAS;[57])	Measures of functional and social impairment were measured using items 2, 3, 4 and 5 (‘home management’, ‘social activities’, ‘private leisure activities’ and ‘close relationships’, respectively) of the WSAS. Item 1 (‘Ability to work’) was not considered in the current analysis as it is routinely scored “N/A" for those not in employment, hence It would have introduced additional bias as many students would likely consider themselves to not currently be in employment/would not be employed outside their studies.

*Demographics and other baseline variables*		
Demographics	–	The dataset also included gender and age when referred, index of multiple deprivation decile, sexual orientation and ethnicity (using the UK census codes ‘White’, ‘Mixed’, ‘Asian’, ‘Black’, ‘Chinese’ and ‘other), all of which were self-reported.
Long-term health conditions	–	Participants also reported whether they had long-term physical health conditions, although the specific nature of reported conditions were not available.
Medication	–	Psychotropic medication use, recorded as prescribed but not taking, prescribed and taking, or not prescribed.
Employment status	–	All participants are asked to report their current employment status. Possible responses included ‘Employed’, ‘Unemployed’, ‘Student’, ‘Long-term sick’, ‘Homemaker’, ‘Not seeking work’, ‘Volunteer’, ‘Retired’. In the current analysis responses of “student” and “employed” only were considered.

*Treatment factors*		
Number of low and high intensity sessions	–	The number of sessions of each type (High intensity: face-to-face, mainly one-to-one (with some group work) sessions with a suitably trained therapist; low intensity: treatments with less intensive therapist input, e.g. guided self-help or computer-based CBT) received during the course of treatment were recorded
Time to assessment	–	Weeks between referral and first assessment⁎
Time to treatment	–	Weeks between assessment and the first treatment session⁎
Length of episode	–	Weeks between assessment and the final treatment session⁎
Service	–	The mental health service the patient was seen at

⁎ converted to weeks from days and winsorized at the top 99% due to a small number of extreme values.

#### 2.3.1 Outcomes

One primary and three secondary dichotomous outcomes were included in the analysis and were defined as follows [29,35]:

##### 2.3.1.1 Primary outcome: reliable recovery

Reliable recovery is used for national evaluations and monitoring of IAPT services [32,34] and is defined as transitioning from ‘caseness’ to ‘non-caseness’ following treatment, and reporting reliable improvement, as defined below (see Table 1 for detail on measures and thresholds).

##### 2.3.1.2 Secondary outcomes

###### 2.3.1.2.1 Reliable Improvement

Reporting a reduction in symptom scores which is more than the reliable change threshold for either the PHQ-9 or GAD-7 (or appropriate anxiety disorder specific measures [ADSM]).

###### 2.3.1.2.2 Deterioration

Reporting an increase in symptom scores which is more than the reliable change threshold for either the PHQ-9 or GAD-7, (or appropriate anxiety disorder specific measures [ADSM]).

###### 2.3.1.2.3 Attrition

Reported as having “dropped out” of the episode of care before completing the planned number of treatment sessions. Only participants who received at least 3 treatment sessions and were not referred on for further care were included for this outcome (9.62% excluded).

### 2.4 Data analysis

#### 2.4.1 Sample characteristics and group differences

Students and same-age employed adults meeting inclusion criteria were first compared on baseline and treatment characteristics to establish what differences existed between students and same-age employed adults attending the services. Independent *t*-tests were conducted to compare differences in means for continuous variables and chi-square tests were conducted to compare categorical variables.

#### 2.4.2 Associations of student status with outcomes

Next, logistic regression models were built to explore the association between student status and outcomes, while controlling for confounders available within the dataset. For each of the four outcomes listed above, the following models were constructed:

**Model 1:** The association of student status (“student” vs “employed”) with outcomes without additional confounders (i.e. unadjusted).

**Model 2:** As in Model 1, additionally controlling for treatment-related variables (number of low intensity sessions, number of high intensity sessions, weeks between referral and assessment, weeks between assessment and treatment, and service.)

**Model 3:** As in Model 2, additionally controlling for baseline symptom and social functioning questionnaire scores (PHQ-9, GAD-7, WSAS2, WSAS3, WSAS4, WSAS5, the three IAPT phobia scale items).

**Model 4:** As in Model 3, additionally controlling for other demographic and clinical factors (IMD decile, age, gender, sexual orientation, ethnicity, problem descriptor, presence of long-term health conditions, medication prescription).

##### 2.4.2.1 Missing data

Missing data on all included continuous variables were imputed using multiple imputations with chained equations (MICE) in Stata 16 [36]. This method was chosen because it is flexible in handling different types of variables and performs well with large datasets [37]. Missing categorical variables were not imputed- these were given a “missing” code to allow participants with missing information on these variables to be included in analyses without removal by list-wise deletion. Fifty imputed datasets were created and imputed data were used for all regression analyses. Sensitivity analyses were run including only complete data.

#### 2.4.3 Propensity score matching

Propensity score matching [38] was conducted to identify matched students to employed younger adults to establish whether student outcomes differed when individuals were similar on all available confounding variables. Model 4 was then replicated using this sample for each outcome. Matching was performed on all variables planned to be entered into the regression models, using “psmatch2” [39] in Stata. Only cases which had complete data for continuous variables were included, however missing categorical data was coded as “missing” as described above. The caliper was set at 0.001 as in previous analyses of this dataset as this has been established as an acceptable level for matching [35]. The first nearest neighbour was identified for each student, meaning the same control case could be identified as the best match for two different student cases, following previous methods using these types of data [35,40]. Cases were weighted according to the number of matches in the analysis.

#### 2.4.4 Treatment moderators

The following moderators were examined:

##### 2.4.4.1 Main treatment intensity

Defined as “high intensity” for individuals where the number of high intensity sessions were more than two and the number of low intensity sessions was less than two, and defined as “low intensity” when the number of low intensity sessions were more than two and the number of high intensity sessions were less than two. Participants whose sessions did not meet either of these criteria were excluded from the analysis, as these individuals may have received a range of treatments.

##### 2.4.4.2 Main treatment medium

Defined as “face to face” if more than half of the total treatment sessions were provided face to face, and “other” if more than half of the total treatment sessions were provided via other formats (this was usually via telephone but in some instances, particularly for those that received high intensity therapy in 2020 was via video–call [41]).

##### 2.4.4.3 Treatment rate

Treatment rate was calculated as the average number of sessions per week. This was calculated by dividing the number of treatment sessions by the length of the treatment episode in weeks.

Interaction terms were fitted in fully adjusted models (Model 4) to explore the effects of moderators. Imputed data were used, and sensitivity analyses were also conducted including only complete data. Models exploring the interaction of main treatment intensity and student status adjusted for the number of sessions in total (instead of the number of high intensity and low intensity sessions separately). Since treatment rate is likely to differ significantly between those who received mainly high intensity sessions and those who received mainly low intensity sessions, analyses were conducted separately for these two groups, and participants whose sessions did not meet criteria for either mainly high intensity or mainly low intensity (29.6%) were excluded from the analysis. In this analysis, students who received less than 4 sessions were also excluded (a further 9.6% of those remaining) in order to create a treatment rate variable that accurately reflected frequency of sessions without being skewed by those who were assessed and subsequently dropped out.

## 3 Results

### 3.1 Baseline differences between students and same-age employed adults

Of 19,707 participants meeting inclusion criteria, 6969 (35%) were students aged between 17 and 25 years old. A participant flow diagram is presented in Appendix A, and Table 2 presents comparisons of baseline and treatment characteristics.

**Table 2:** Baseline differences between students and employed adults aged 17–25.

	Students			Employed				
	n	M	SD	n	M	SD	t	p
PHQ-9	6968	15.11	5.25	12,738	14.49	5.35	7.87	<0.001
GAD-7	6967	13.61	4.27	12,736	13.86	4.24	−3.95	<0.001
WSAS-item 2	5785	3.38	2.32	10,999	3.44	2.32	1.06	0.291
WSAS-item 3	5785	4.38	2.62	10,999	4.24	2.25	3.84	<0.001
WSAS-item 4	5783	3.55	2.42	10,996	3.48	2.46	1.79	0.074
WSAS-item 5	5785	4.15	2.36	10,995	4.16	2.38	−0.10	0.919
Agoraphobia item	6873	2.85	2.59	12,548	2.63	2.57	5.57	<0.001
Social phobia item	6872	3.44	2.42	12,557	3.07	2.39	10.22	<0.001
Specific phobia item	6872	2.29	2.55	12,551	2.03	2.50	6.80	<0.001
Number LI sessions	6969	2.70	2.63	12,738	2.80	2.62	−2.66	0.008*
Number HI sessions	6969	4.56	5.31	12,738	4.75	5.49	−2.32	0.020
Weeks - referral to assessment	6964	3.46	3.36	12,734	3.35	3.48	2.04	0.041
Weeks - assessment to treatment	6540	7.94	7.83	11,963	8.63	8.30	−5.65	<0.001
Age	6969	20.72	2.21	12,738	22.98	1.91	−71.75	<0.001


		Students		Employed			
		N	%	n	%	X^2^	p
Gender	Male	1818	26.09%	3433	26.95%	1.73	0.192
	Female	5102	73.21%	9218	72.37%		
	Missing	49	0.70%	87	0.68%		
Ethnicity	White	3577	51.33%	8317	65.29%	489.73	<0.001
	Mixed	587	8.42%	950	7.46%		
	Asian	1127	16.17%	1179	9.26%		
	Black	796	11.42%	1243	9.76%		
	Chinese	149	2.14%	94	0.74%		
	Other	288	4.13%	273	2.14%		
	Missing	445	6.39%	682	5.35%		
IMD decile	1	586	8.41%	1097	8.61%	34.89	<0.001
	2	1800	25.83%	3422	26.86%		
	3	1464	21.01%	2694	21.15%		
	4	872	12.51%	1621	12.73%		
	5	772	11.08%	1128	8.86%		
	6	538	7.72%	1007	7.91%		
	7	349	5.01%	610	4.79%		
	8	269	3.86%	599	4.70%		
	9	129	1.85%	235	1.84%		
	10	49	0.70%	98	0.77%		
	Missing	141	2.02%	227	1.78%		
Sexual orientation	Heterosexual	4508	64.69	8209	64.44%	19.46	<0.001
	Gay/Lesbian	218	3.13	377	2.96		
	Bi-sexual	371	5.32	523	4.11		
	Missing	1872	26.86	3629	28.49		
Medication	Prescribed - not taking	603	8.65%	916	7.19%	21.95	<0.001
	Prescribed and taking	2094	30.05%	3709	29.12%		
	Not prescribed	3931	56.41%	7378	57.92%		
	Missing	341	4.89%	735	5.77%		
Long term condition	No	4782	68.62%	8652	67.92%	5.43	0.066
	Yes	958	13.75%	1677	13.17%		
	Missing	1229	17.64%	2409	18.91%		
Problem descriptor	Depression	2314	33.20%	4388	34.45%	71.61	<0.001
	Mixed A.D.	326	4.68%	646	5.07%		
	GAD	929	13.33%	2006	15.75%		
	OCD	213	3.06%	312	2.45%		
	PTSD	151	2.17%	277	2.17%		
	Other phobia and panic	330	4.74%	767	6.02%		
	Social phobia	381	5.47%	598	4.69%		
	Unspecified anxiety	268	3.85%	481	3.78%		
	Missing	2057	29.52%	3263	25.62%		
Clinical outcomes recorded	Reliable recovery	2920	41.90%	6086	47.78%	62.73	<0.001
	Reliable improvement	4678	67.13%	9138	71.74%	45.72	<0.001
	Deterioration	501	7.19%	840	6.59%	2.51	0.113
	Drop-out	2141	34.25%	3770	32.61%	4.91	0.027

Note. WSAS: Work and social adjustment scale items. LI: low intensity HI: high intensity.

University students had higher scores on measures of depression (*p* < .001), but lower scores on measures of anxiety at baseline (p < .001). They also had higher scores on the measures for specific phobia (*p* < .001) and social phobia (p < .001) but lower agoraphobia scores (p < .001). However, fewer students had depression or generalized anxiety as their recorded problem descriptor (diagnosis), with more OCD and phobias recorded. Students also reported more impairment in social leisure activities (p < .001), although other WSAS scale item scores did not appear to differ between students and non-students. Mean numbers of both low intensity and high intensity sessions were lower in students (*p* = .008 & *p* = .020, respectively) and they experienced elevated waiting times from referral to assessment (*p* = .041). However, they experienced reduced waiting times from assessment to treatment (*p* < .001). Although the cohort included more females than males, the balance was similar between students and employed adults (*p* = .192), as was the presence of long-term health conditions (*p* = .066). The sample was limited to ages 17–25, but the mean age of students was lower than that of employed adults (*p* < .001). There were significant differences in ethnicity between the two groups (p < .001); the student group encompassed more ethnic minority participants compared to the employed group, although in both the majority of participants described their ethnicity as ‘White’.

The percentages of students and employed adults who experienced reliable recovery, reliable improvement, deterioration and attrition are also shown in Table 2. Overall, fewer students reliably recovered (41.9% vs 47.8%; *p* < .001) and reliably improved (67.1% vs 71.7%; p < .001), and more students dropped out (34.3% vs 32.6%; *p* = .027). Similar proportions of students and non-students reliably deteriorated (7.2% vs 6.6%; *p* = .113).

### 3.2 The association of student status with clinical outcomes

Table 3 shows the results of logistic regression models exploring associations between student status and outcomes. After adjusting for number of sessions attended, waiting times and the service attended, students (vs employed) were less likely to reliably recover (OR = 0.82, 95% CI: 0.76–0.86) and reliably improve (OR = 0.83, 95% CI: 0.78–0.89). Attrition was more likely in students (OR = 1.12, 95% CI: 1.04–1.21) but there was no evidence of a difference in the odds of deterioration (OR = 1.07, 95% CI: 0.95–1.20). After also controlling for baseline severity, students continued to be less likely to reliably recover (OR = 0.85, 95% CI: 0.80–0.90) and reliably improve (OR = 0.85, 95% CI: 0.80–0.91), though the association between student status and attrition was no longer significant (OR = 1.07, 95% CI: 0.99–1.16). Controlling for all service level, baseline symptom and demographic variables, students were less likely to reliably recover (OR = 0.90, 0.83–0.96) and reliably improve (OR = 0.91, 95% CI: 0.84–0.98). There remained no evidence that students were more likely to deteriorate (OR = 0.89, 95% CI: 0.78–1.02) or drop out (OR = 1.01, 95% CI: 0.93–1.11) than same-age employed adults. Sensitivity analyses conducted on complete cases only showed similar results (Appendix B).

**Table 3:** Logistic regression models of the association between student status and outcomes.

		Reliable Recovery	Reliable Improvement	Deterioration	Attrition
Model 1	Student	0.78 (0.74–0.84)	0.80 (0.76–0.86)	1.10 (0.98–1.23)	1.08 (1.01–1.15)
Model 2	+ Service level variables ⁎	0.82 (0.76–0.86)	0.83 (0.78–0.89)	1.07 (0.95–1.20)	1.12 (1.04–1.21)
Model 3	+ Baseline severity ǂ	0.85 (0.80–0.90)	0.85 (0.80–0.91)	1.03 (0.91–1.16)	1.07 (0.99–1.16)
Model 4	+ Demographic factors §	0.90 (0.83–0.96)	0.91 (0.84–0.98)	0.89 (0.78–1.02)	1.01 (0.93–1.11)
Model 4 matched	Matched controls⸹	0.87 (0.80–0.94)	0.85 (0.78–0.93)	0.88 (0.75–1.03)	0.96 (0.86–1.06)

⁎ Number low intensity sessions, number high intensity sessions, weeks between referral and assessment, weeks between assessment and treatment, trust.

ǂ PHQ9, GAD7, Work and Social Adjustment Scale items 2–5, phobias [55,57,61].

§ IMD, age, gender ethnicity, diagnosis, long term conditions, medication use, sexual orientation.

⸹ *N* = 10,640 for reliable recovery, reliable improvement and deterioration. *N* = 9789 for attrition.

### 3.3 Matching

Propensity score matching was performed including all baseline variables. Acceptable matches were not found for 93 students. Once these cases were excluded, 5320 students and matched non-student controls were included in the analyses. Comparisons o f baseline characteristics between students and their matched controls were conducted (See Appendix C). Good balance was achieved, with small but significant differences found only for age and ethnicity. Results of the regression analysis using this sample were conducted on complete cases only and are also displayed in Table 3. After matching, students were less likely to reliably recover (OR = 0.87, 95% CI: 0.80–0.94) and reliably improve (OR = 0.85, 95% CI: 0.78–0.93), and there was no evidence of differences in the odds of deterioration (OR = 0.88, 95%: 0.75–1.03) or attrition (OR = 0.96, 95% CI: 0.86–1.06).

### 3.4 Moderators of outcomes

**Fig. 1:**
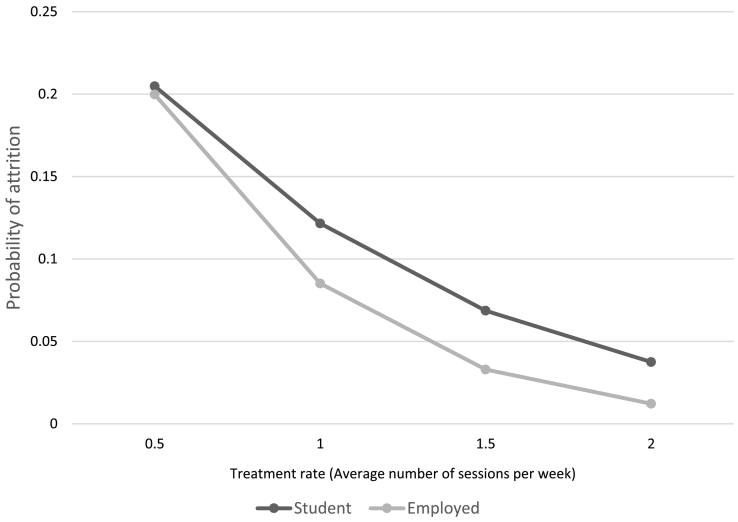
Moderation of the association between student status and attrition in those receiving mainly high intensity treatments by treatment rate.

There was no evidence that the main intensity of treatment received, or the main modality of treatment (face to face or telephone) moderated the effect of student status on outcomes (e.g. for reliable recovery, *p* = .088 and *p* = .745, respectively; see Appendix D). There was also no evidence that treatment rate moderated outcomes of reliable recovery, reliable improvement or deterioration. However, in the mainly high intensity sub-group treatment rate significantly moderated the effect of student status on attrition, such that students experienced less improvement in the likelihood of dropout with increasing frequency of sessions compared to employed adults. In the mainly low intensity subgroup treatment rate did not moderate attrition (see Appendix E and Fig. 1).

## 4 Discussion

This study aimed to explore the differences in outcomes between university students aged 17–25 and employed people of the same age receiving therapy from external mental health services. Students had reduced odds of experiencing positive outcomes such as reliable recovery or reliable improvement, but were equally as likely to experience a reliable deterioration or to drop out of treatment compared to employed controls. These findings were similar across the whole sample and when analysing a propensity-score matched sample, and were not moderated by treatment specific variables such as waiting times, intensity, frequency or medium of treatment, suggesting a good degree of robustness.

Previous research has suggested that reports of poor student mental health may be the product of factors relating to emerging adulthood [3,9]. However, the results of this analysis suggest that in considering treatment outcomes, age is not a key factor in determining poor outcomes in students; even when controlling for age, students are less likely to have positive treatment outcomes compared to their non-student peers. One possible explanation is that a factor unmeasured within the current dataset relevant to the context of being a student reduces the effectiveness of psychological treatment. For example, many have argued that a lack of strong social support is particularly common in university students [9,42] due to the requirement to form new relationships, in new social settings when attending university away from home [43], which, combined with increasing academic demands can mean that some students fail to form a new social support network [9]. This has been linked to poor mental health outcomes in this population [7]. Although we controlled for measures of social functioning, it is likely that this does not capture in totality the impact of the social dislocation inherent in moving away from home to attend higher education. A second possibility is that disruptions to IAPT treatment may occur for students during holiday or exam periods- students had significantly fewer treatment sessions compared to non-students and this is an established factor impacting treatment outcomes in IAPT [29]. However, adjusting for the number of high and low intensity sessions received did not impact on results, and treatment rate (which would be lower for large episodes of absence during holidays) showed limited impact here.

It has been suggested that students are more likely to drop out of interventions [25], but this was not supported by the current study. A qualitative study reported that students want more, rather than less treatment [18], and this may particularly impact attrition in those students who seek additional support external to university services. Of note, in those receiving predominantly high intensity care, students may not experience a reduced likelihood of attrition with higher session frequency to the extent that employed adults do.

### 4.1 Limitations

In considering the results of this study, some important limitations should be considered. Students and their same aged employed peers were selected from the dataset using a self-report employment variable, which was worded so that the “student” response required the participant to be not working or seeking employment. As there is only one response allowed on this question, we may have missed an important group of students who work alongside their studies and have therefore identified as employed. This is most likely among students who study part-time, particularly common among post-graduate students, who arguably may have more stressors such as additional work and family commitments alongside study [44]. Although this makes our estimates conservative as the effect may have been weakened by comparison to a group which may contain some of these students, consideration of post-graduate and part-time students is important for future work, as mental health problems are highly prevalent in this population [45]. Similarly, the dataset is also limited by the lack of contextual information regarding academic or other higher education factors, which prevents definitive examination of the hypothesis that these may influence outcomes. Future research should look to explore the association between such factors and treatment outcomes in students.

In addition, although we adjusted for all available confounding variables and used propensity score matching as an alternative way to deal with confounding, we cannot rule out residual confounding by factors unable to be adjusted for here, for example the age of initial onset of mental health problems [5], which future research should aim to explore further. Propensity score matching also did not remove baseline differences in ethnicity and age between students and employed adults. We limited our sample only to those aged between 17 and 25 years old, and there could be some differences in responses to treatment at different ages within this group. A recent systematic review and individual patient data meta-analysis has however found that age is not associated with treatment outcomes for adults with depression treated in primary care [46]. Students from minority ethnic groups could be considered particularly at risk of poor mental health outcomes [47], and as such this may have contributed to the poorer outcomes in the student group. Controlling for age and ethnicity did not remove the association of student status and recovery or improvement, however, supporting our assertion that there is something inherent in being a university student which is associated with poorer odds of recovery post-treatment.

### 4.2 Conclusions and implications

The results of this study suggest that students have poorer outcomes from psychological therapy in mental health care settings external to university. As such, it may be important to consider adapting care pathways and the content of interventions to meet student-specific needs [48]. In line with recent calls for the integration of local external mental health services with university based services, and for all services and communications about these services to be adapted so that they are made relevant for students [23], such an approach may contribute to improved outcomes for students being seen within routine psychological services. For example, services might offer additional support for highly prevalent co-occurring stressors such as academic/exam stress [6,7], financial stress [10] and lack of social support [6,9,14] to combat potential barriers to recovery. Where guidance on adaptation exists (e.g. for alcohol and substance use; [13]), further consideration of how to make such support more accessible to students may be required. Social support in particular is often relied upon by young people when seeking support for mental health problems [49] and as such could boost effects of both university-based and external mental health care. Moreover, similarly to specific staff training for children and young people [50], it is likely that staff working in mental health services would welcome additional training in supporting students, particularly given that there is an association between the proportion of experienced staff and positive outcome [51]. Finally, disruptions in treatment resulting from term-time and holiday living arrangements may necessitate that specific additional considerations are taken into account in planning treatment for students. This could include prioritising integration between routine health care services and university mental health services to allow for periodic changes in living location and perhaps encompassing remotely delivered care such as video-conferencing which has become more readily used in services since the start of the COVID-19 pandemic [41,52].

## Contributors

PB contributed to the study design, conducted analyses and drafted the original manuscript and appendices. RS contributed to the study design, analysis plan, data interpretation, supported the drafting of the manuscript and final approval. JB contributed to the study design, analysis plan, data interpretation, supported the drafting of the manuscript and final approval. AC contributed to the data interpretation, drafting of the manuscript and final approval. MC contributed to the data interpretation, drafting of the manuscript and final approval. JL contributed to the data interpretation, drafting of the manuscript and final approval. NM contributed to the data interpretation, drafting of the manuscript and final approval. SN contributed to the data interpretation, drafting of the manuscript and final approval. SS contributed to the data interpretation, drafting of the manuscript and final approval. JS contributed to the data interpretation, drafting of the manuscript and final approval. LV contributed to the data interpretation, drafting of the manuscript and final approval. JW contributed to the data interpretation, drafting of the manuscript and final approval. SP contributed to the study design, analysis plan, data interpretation, supporting the drafting of the manuscript and final approval.

## Role of the funding source

S.P. was supported by the 10.13039/501100012317University College London Hospitals Biomedical Research Centre, 10.13039/501100000765University College London and the 10.13039/100010248Royal College of Psychiatrists.

J.E.J.B. was supported by the 10.13039/100010269Wellcome Trust (grant no: 201292/Z/16/Z), the 10.13039/501100000272National Institute for Health Research 10.13039/501100012317University College London Hospitals Biomedical Research Centre and the 10.13039/100010248Royal College of Psychiatrists.

JS was supported by the 10.13039/501100012317University College London Hospitals Biomedical Research Centre, 10.13039/501100000765University College London (grant no: 696100), a grant from Alzheimer's Society (457(AS-PG-18-013)), (236 (AS-CTF-14-005) a grant from ESRC/NIHR funding initiative (ES/S010467/1); a grant from 10.13039/501100000377Dunhill medical trust RPGF1910\191 and a grant from the NIHR (NIHR130914).

The funding sources had no role in the study design, data collection, analysis, interpretation of data or manuscript preparation.

## Declaration of Competing Interest

There are no conflicts of interest for any authors involved in the preparation of this review.

## Appendix A Supplementary data

Supplementary material Image 1
