# Supplementary material for: Are students less likely to respond to routinely delivered psychological treatment? A retrospective cohort analysis
Source: Compr Psychiatry. 2022 Nov;119:152348. doi: 10.1016/j.comppsych.2022.152348 (PMC9760567; doi:10.1016/j.comppsych.2022.152348)
Supplement: Supplementary file 1 — Supplementary material [file mmc1.docx]

**Supplementary Material**

**Appendix A: Participant flow diagram**

483,683 original referrals

99,621 participants remaining

Age outside 17-25 at first assessment

<17: 1,930 participants excluded

>25: 382,103 participants excluded

Age missing: 29 participants excluded

Employment status not marked as either employed or student (e.g. homemaker, retired, unemployed): 58,986 participants excluded

40,635 participants remaining

Did not enter treatment (at least two treatment sessions): 18,945 participants excluded

21,690 participants remaining

Not scoring above the cut off for “caseness” of anxiety or depression: 1,983 participants excluded

19,707 participants remaining

Employed: 12,738

Student: 6,969

| **Appendix B: Logistic regression analysis using complete cases only** | | | | | |
| --- | --- | --- | --- | --- | --- |
|  |  | **Reliable Recovery** | **Reliable Improvement** | **Deterioration** | **Attrition** |
| **Model 1** | Student | 0.78  (0.74-0.84) | 0.80  (0.76-0.86) | 1.10  (0.98-1.23) | 1.08  (1.01-1.15) |
| **Model 2** | + Service level variables * | 0.80  (0.75-0.85) | 0.83  (0.77-0.89) | 1.09  (0.96-1.23) | 1.12  (1.04-1.21) |
| **Model 3** | + Baseline severity ǂ | 0.83  (0.78-0.90) | 0.86  (0.79-0.92) | 1.04  (0.90-1.19) | 1.10  (1.01-1.20) |
| **Model 4** | + Demographic factors § | 0.88  (0.81-0.95) | 0.90  (0.82-0.98) | 0.91  (0.78-1.07) | 1.06  (0.95-1.17) |
| * Number low intensity sessions, number high intensity sessions, weeks from referral to assessment, weeks from assessment to treatment, service. | | | | | |
| ǂPHQ9, GAD7, Work and Social Adjustment Scale items 2-5, phobias | | | | | |
| § IMD, age, gender ethnicity, diagnosis, long term conditions, medication use, sexual orientation. | | | | | |
|  | | | | | |

| **Appendix C: Balance between students and matched controls.** | | | | | | | |
| --- | --- | --- | --- | --- | --- | --- | --- |
| **Patient characteristic** | | **Student** | | **Same age employed** | | **t** | **p** |
|  |  | **Mean** | **SD** | **Mean** | **SD** |  |  |
| PHQ-9 | | 15.12 | 5.21 | 14.97 | 5.30 | 1.23 | .220 |
| GAD-7 | | 13.63 | 4.30 | 13.72 | 4.30 | -0.88 | .378 |
| WSAS-2 | | 3.38 | 2.32 | 3.31 | 2.34 | 1.40 | .163 |
| WSAS-3 | | 4.38 | 2.26 | 4.39 | 2.28 | -0.23 | .817 |
| WSAS-4 | | 3.54 | 2.41 | 3.51 | 2.51 | 0.44 | .658 |
| WSAS-5 | | 4.15 | 2.35 | 4.17 | 2.41 | -0.40 | .687 |
| Agoraphobia item | | 2.95 | 2.60 | 2.94 | 2.67 | 0.11 | .913 |
| Social phobia item | | 2.53 | 2.41 | 3.48 | 2.49 | 0.92 | .356 |
| Specific phobia item | | 2.35 | 2.57 | 2.27 | 2.60 | 1.34 | .182 |
| Number HI sessions | | 4.90 | 5.32 | 4.78 | 5.35 | 0.92 | .357 |
| Number LI sessions | | 2.84 | 2.74 | 2.88 | 2.74 | -0.58 | .563 |
| Waiting time (weeks) - referral to assessment | | 3.35 | 3.29 | 3.25 | 3.62 | 1.29 | .197 |
| Waiting time (weeks) - assessment to treatment | | 8.35 | 7.94 | 8.48 | 7.90 | -0.67 | .503 |
| Age | | 20.68 | 2.19 | 21.51 | 2.09 | -16.91 | <.001 |
| **Patient characteristic** | | **Student** | | **Employed** | |  | |
|  |  | **n** | **%** | **n** | **%** | **X^2^** | **P** |
| Gender | Male | 1368 | 25.71 | 730 | 26.05 | 0.18 | .915 |
|  | Female | 3933 | 73.93 | 2061 | 73.55 |  |  |
|  | Missing | 19 | 0.36 | 11 | 0.39 |  |  |
| Ethnicity | White | 2711 | 50.96 | 1566 | 55.89 | 31.99 | <.001 |
|  | Mixed | 460 | 8.65 | 224 | 7.99 |  |  |
|  | Asian | 933 | 17.54 | 402 | 14.35 |  |  |
|  | Black | 649 | 12.20 | 374 | 13.35 |  |  |
|  | Chinese | 95 | 1.79 | 39 | 1.39 |  |  |
|  | Other | 213 | 4.00 | 90 | 3.21 |  |  |
|  | Missing | 259 | 4.87 | 107 | 3.82 |  |  |
| Index of Multiple Deprivation (IMD) Decile | 1 | 447 | 8.44 | 251 | 8.84 | 3.89 | 0.952 |
|  | 2 | 1373 | 25.92 | 759 | 26.73 |  |  |
|  | 3 | 1108 | 20.91 | 566 | 19.93 |  |  |
|  | 4 | 636 | 12.00 | 340 | 11.97 |  |  |
|  | 5 | 569 | 10.74 | 301 | 10.60 |  |  |
|  | 6 | 426 | 8.04 | 227 | 7.99 |  |  |
|  | 7 | 287 | 5.42 | 143 | 5.04 |  |  |
|  | 8 | 220 | 4.15 | 124 | 4.37 |  |  |
|  | 9 | 104 | 1.96 | 51 | 1.80 |  |  |
|  | 10 | 43 | 0.81 | 23 | 0.81 |  |  |
|  | Missing | 85 | 1.60 | 55 | 1.94 |  |  |
| Sexual Orientation | Heterosexual | 3683 | 69.23 | 1942 | 69.31 | 2.13 | .546 |
|  | Gay/Lesbian | 178 | 3.35 | 91 | 3.25 |  |  |
|  | Bi-sexual | 299 | 5.62 | 138 | 4.93 |  |  |
|  | Missing | 1160 | 21.80 | 631 | 22.52 |  |  |
| Medication prescribed | Prescribed | 1553 | 29.19 | 831 | 29.66 | 2.58 | .275 |
|  | Not prescribed | 3475 | 65.32 | 1795 | 64.06 |  |  |
|  | Missing | 292 | 5.49 | 176 | 6.28 |  |  |
| LTC Case | No | 3578 | 67.26 | 1897 | 67.70 | 0.37 | .830 |
|  | Yes | 798 | 15.00 | 423 | 15.10 |  |  |
|  | Missing | 944 | 17.74 | 482 | 17.20 |  |  |
| Problem descriptor | Depression | 2108 | 39.62 | 1115 | 39.79 | 3.63 | .889 |
|  | GAD | 869 | 16.33 | 481 | 17.17 |  |  |
|  | Mixed A.D | 287 | 5.39 | 149 | 5.32 |  |  |
|  | OCD | 188 | 3.53 | 86 | 3.07 |  |  |
|  | Other Phobia and Panic | 308 | 5.79 | 168 | 6.00 |  |  |
|  | PTSD | 137 | 2.58 | 78 | 2.78 |  |  |
|  | Social Phobia | 348 | 6.54 | 169 | 6.03 |  |  |
|  | Unspecified Anxiety | 250 | 4.70 | 122 | 4.35 |  |  |
|  | Missing | 825 | 15.51 | 434 | 15.49 |  |  |
| *Note.* SD: Standard deviation WSAS: Work and Social Adjustment Scale A.D: Anxiety Disorders | | | | | | | |

| **Appendix D.1: Associations between each outcome and student status moderated by treatment intensity and modality in fully adjusted models^*^ (Imputed data)** | | | | |
| --- | --- | --- | --- | --- |
| **Interaction** | **Reliable Recovery** | **Reliable Improvement** | **Reliable Deterioration** | **Attrition** |
| Student by main intensity (high intensity) ǂ | 0.88  (0.76-1.02) | 0.92  (0.78-1.09) | 0.83  (0.62-1.13) | 1.19  (0.98-1.44) |
| Student by main modality (face to face) § | 0.97  (0.83-1.14) | 0.92  (0.78-1.09) | 1.07  (0.78-1.47) | 0.99  (0.82-1.19) |
| ^*^All models adjusted for number of sessions, weeks from referral to assessment, weeks from assessment to treatment, service, PHQ-9 scores, GAD-7 scores, Work and Social Adjustment Scale item 2-5 scores, IAPT phobias scale item scores, IMD, age, gender, ethnicity, diagnosis, long term conditions, medication use, and sexual orientation  ǂ N= 13,489 for reliable recovery, reliable improvement and deterioration. N=12,607 for attrition.  § N= 17,411 for reliable recovery, reliable improvement and deterioration. N=16,105 for attrition.  *Note.* IAPT: Improving Access to Psychological Therapies. IMD: Index of Multiple Deprivation. | | | | |

| **Appendix D.2: Associations between each outcome and student status moderated by treatment intensity and modality in fully adjusted models^*^ using complete cases only** | | | | |
| --- | --- | --- | --- | --- |
| **Interaction** | **Reliable Recovery** | **Reliable Improvement** | **Reliable Deterioration** | **Attrition** |
| Student by main intensity (high intensity) ǂ | 0.92 (0.78-1.08) | 0.92 (0.77-1.11) | 0.87 (0.62-1.22) | 1.13 (0.92-1.40) |
| Student by main modality (face to face) § | 0.96 (0.82-1.13) | 0.92 (0.77-1.10) | 1.11 (0.80-1.54) | 0.99 (0.81-1.20) |
| ^*^All models adjusted for number of sessions, weeks from referral to assessment, weeks from assessment to treatment, service, PHQ-9 scores, GAD-7 scores, WSAS items 2-5 scores, IAPT phobias scale item scores, IMD, age, gender, ethnicity, diagnosis, long term conditions, medication use, and sexual orientation  ǂ N= 11,530 for reliable recovery, reliable improvement and deterioration. N=10,825 for attrition.  § N= 14,742 for reliable recovery, reliable improvement and deterioration. N=13,710 for attrition.  *Note.* IAPT: Improving Access to Psychological Therapies. IMD: Index of Multiple Deprivation. | | | | |

| **Appendix E.1: Associations between each outcome and student status moderated by treatment rate fully adjusted models^*^, by main intensity type (Imputed data)** | | | | |
| --- | --- | --- | --- | --- |
| **Interaction** | **Reliable Recovery** | **Reliable Improvement** | **Reliable Deterioration** | **Attrition** |
| Student by treatment rate (Mainly high intensity sub-group) ǂ | 0.74  (0.46-1.19) | 1.24  (0.72-2.14) | 0.44  (0.15-1.30) | 2.12  (1.11-4.03) |
| Student by treatment rate (Mainly low intensity sub-group) § | 0.84  (0.50-1.40) | 0.99  (0.56-1.78) | 1.08  (0.35-3.33) | 0.64  (0.34-1.21) |
| ^*^All models adjusted weeks from referral to assessment, weeks from assessment to treatment, service, PHQ-9 scores, GAD-7 scores, WSAS items 2-5 scores, IAPT phobias scale item scores, IMD, age, gender, ethnicity, diagnosis, long term conditions, medication use, and sexual orientation  ǂ N= 6930 for reliable recovery, reliable improvement and deterioration. N=6560 for attrition.  § N= 5317 for reliable recovery, reliable improvement and deterioration. N=4977 for attrition.  *Note.* IAPT: Improving Access to Psychological Therapies. IMD: Index of Multiple Deprivation. | | | | |
| **Appendix E.2: Associations between each outcome and student status moderated by treatment rate fully adjusted models^*^, by main intensity type using complete cases only** | | | | |
| **Interaction** | **Reliable Recovery** | **Reliable Improvement** | **Reliable Deterioration** | **Attrition** |
| Student by treatment rate (Mainly high intensity sub-group) ǂ | 0.61  (0.37-1.01) | 1.13  (0.63-2.04) | 0.36  (0.11-1.18) | 2.04  (1.03-4.04) |
| Student by treatment rate (Mainly low intensity sub-group) § | 0.74  (0.43-1.29) | 0.94  (0.50-1.78) | 1.07  (0.29-3.89) | 0.66  (0.33-1.31) |
| ^*^All models adjusted weeks from referral to assessment, weeks from assessment to treatment, service, PHQ-9 scores, GAD-7 scores, WSAS items 2-5 scores, IAPT phobias scale item scores, IMD, age, gender, ethnicity, diagnosis, long term conditions, medication use, and sexual orientation  ǂ N= 6,110 for reliable recovery, reliable improvement and deterioration. N=5,782 for attrition.  § N= 4,529 for reliable recovery, reliable improvement and deterioration. N=4264 for attrition.  *Note.* IAPT: Improving Access to Psychological Therapies. IMD: Index of Multiple Deprivation. | | | | |
